# Development of visual cortical function in infant macaques: A BOLD fMRI study

**DOI:** 10.1371/journal.pone.0187942

**Published:** 2017-11-16

**Authors:** Tom J. Van Grootel, Alan Meeson, Matthias H. J. Munk, Zoe Kourtzi, J. Anthony Movshon, Nikos K. Logothetis, Lynne Kiorpes

**Affiliations:** 1 Max Planck Institute for Biological Cybernetics, Tübingen, Germany; 2 Center for Neural Science, New York University, New York, United States of America; 3 Behavioural and Brain Sciences, School of Psychology, University of Birmingham, Birmingham, United Kingdom; 4 Department of Biology, TU Darmstadt, Darmstadt, Germany; 5 Department of Psychology, University of Cambridge, Cambridge, United Kingdom; Universidad de Salamanca, SPAIN

## Abstract

Functional brain development is not well understood. In the visual system, neurophysiological studies in nonhuman primates show quite mature neuronal properties near birth although visual function is itself quite immature and continues to develop over many months or years after birth. Our goal was to assess the relative development of two main visual processing streams, dorsal and ventral, using BOLD fMRI in an attempt to understand the global mechanisms that support the maturation of visual behavior. Seven infant macaque monkeys (*Macaca mulatta*) were repeatedly scanned, while anesthetized, over an age range of 102 to 1431 days. Large rotating checkerboard stimuli induced BOLD activation in visual cortices at early ages. Additionally we used static and dynamic Glass pattern stimuli to probe BOLD responses in primary visual cortex and two extrastriate areas: V4 and MT-V5. The resulting activations were analyzed with standard GLM and multivoxel pattern analysis (MVPA) approaches. We analyzed three contrasts: Glass pattern present/absent, static/dynamic Glass pattern presentation, and structured/random Glass pattern form. For both GLM and MVPA approaches, robust coherent BOLD activation appeared relatively late in comparison to the maturation of known neuronal properties and the development of behavioral sensitivity to Glass patterns. Robust differential activity to Glass pattern present/absent and dynamic/static stimulus presentation appeared first in V1, followed by V4 and MT-V5 at older ages; there was no reliable distinction between the two extrastriate areas. A similar pattern of results was obtained with the two analysis methods, although MVPA analysis showed reliable differential responses emerging at later ages than GLM. Although BOLD responses to large visual stimuli are detectable, our results with more refined stimuli indicate that global BOLD activity changes as behavioral performance matures. This reflects an hierarchical development of the visual pathways. Since fMRI BOLD reflects neural activity on a population level, our results indicate that, although individual neurons might be adult-like, a longer maturation process takes place on a population level.

## Introduction

The primate visual system is organized in a hierarchical manner [1]. Visual processing by distinct brain areas within this hierarchy has been extensively investigated in adults. However, knowledge about how this organization and area-specific visual processing develops is limited. There is disagreement as to whether development proceeds hierarchically, from primary sensory areas to higher-order ones, or globally with all cortical areas maturing in concert [2–6].

Behavioral measures of visual function reveal extended time periods of development with most visual functions being immature at birth and developing over particular time courses depending on the type of visual ability [7,8]. A clear link between behavioral and neural development has been elusive [9–15]. Our main goal was to relate the development of the visual cortical processing streams to the development of visual function.

Immature animals have lower visual sensitivity for fine detail, contrast, and motion than adults. Although visual behavior in macaques remains immature for many months or even years after birth, anatomically the brain grows to near adult size quite rapidly. Total brain volume begins to asymptote between 3 and 4 months but continues to increase slowly for several years thereafter [16,17]. White matter also increases rapidly initially but slows considerably after the age of 1.5 years [16]. In addition, electrophysiological recordings from early visual areas show that neuronal receptive field organization is surprisingly similar in infants and adults. Receptive field properties are quite mature in primary visual cortex (V1) soon after birth [9,11,18]. Neuronal receptive field structure in visual cortical area V2 is also similar to that of adults by 4 to 8 weeks in infant macaques [19,20]. However, overall response strength is weaker and more sluggish in infants in early visual areas [9,10,12,20]. In general, the data show that the anatomical and physiological organization of the early visual pathways is strikingly mature in comparison to visual behavior. Therefore it is important to look further along the visual pathways for immaturities that may explain the poor vision of infants.

Different visual areas are believed to play different computational roles in the cascade of visual processing. The dorsal and ventral visual processing streams are functionally distinct pathways that, according to the classical literature, divide processes of visual encoding into “where” and “what” respectively [21–23] see [24]. These pathways are thought to represent visual information to answer these questions: *What* is in the visual field, and *where* is it and *how* should an appropriate interaction be assigned. While the segregation of the pathways is more conceptual than real, they channel information differentially to the temporal visual association and parietal visual-motor regions of the brain. Their relative maturation is a matter of some debate [25,26]. A previous psychophysical study on the development of global motion sensitivity, as an assay for dorsal stream function, and global form sensitivity, as an assay for ventral stream function, in infant monkeys showed that both continue to mature over about the first 2 years after birth, although sensitivity to visual motion appears earlier in development than global form perception [26].

Little is known about the functional development of visual areas in these extrastriate pathways. Distler and colleagues used a metabolic assay, 2-DG, to map the relative development of striate and extrastriate brain areas in young macaques [3]. They found evidence for earlier maturation of dorsal stream areas than ventral stream ones. Neurophysiological investigations have found visually responsive, well-tuned neurons in inferior temporal cortex (IT) in the ventral stream and MT in the dorsal stream at the earliest ages recorded (6 weeks and 1 week, respectively) [10,27–29], although Rodman and colleagues noted reduced responsiveness in IT compared with MT in the same animals at young ages. Our previous behavioral study [26] is consistent with the suggestion from these neural data that there is somewhat earlier maturation of dorsal stream, however, it is difficult to draw any strong conclusion given the scarcity of neural data.

So, on the one hand, visual behavior in infants is not comparable to that of adults and continues to develop over years. On the other hand, electrophysiology reveals that the cortical machinery of vision seems relatively adult-like within weeks of birth. To understand this mismatch we sought a more global measure than single neuron electrophysiology. A macro-scale sampling method can be utilized to identify interactions and activation patterns of multiple brain areas simultaneously. Therefore we employed Blood Oxygenation Level Dependent functional Magnetic Resonance Imaging (BOLD fMRI) to track development of the extrastriate visual pathways.

In an earlier study we used fMRI to trace visual system development [30]. In that study, we used large rotating checkerboard stimuli and generalized linear models (GLMs) to identify developmental trends. For a GLM, the BOLD responses are estimated based on the on-off alternation of the stimulus presentation. Correlation of the predicted response during a stimulus is linearly regressed with the measured response. Previously we identified reliable activation at the youngest ages (3 to 5 months) in V1, but there was no apparent activation of downstream, extrastriate areas V4 and MT-V5 in animals younger than 14 months. In the current study we opted to use a Multivoxel Pattern Analyses (or multivariate pattern analyses, MVPA, [31–34] see [35]) approach to uncover signals that might have remained subthreshold in a GLM analysis. MVPA are expected to be more sensitive, since activity patterns are combined, rather than comparing activation within a single voxel.

**Fig 1:**
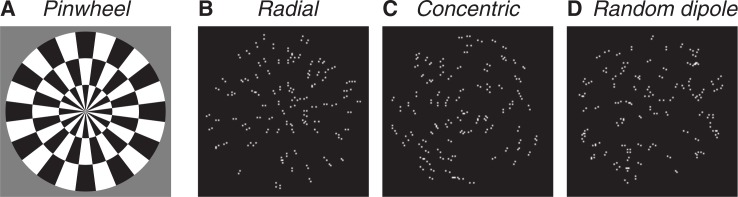
Examples of stimuli. (A) High contrast pinwheel stimulus. Glass patterns (B,C) are based on paired dots (dipoles); structured patterns are formed when the orientation of the dipoles conforms to a particular underlying rule. (B) A radial pattern or (C) a concentric pattern. (D) When there is no underlying rule, a random dipole pattern is created.

We chose to target three visual areas: V1, primary visual cortex; MT-V5 in the dorsal stream which is involved in encoding motion stimuli; with its counterpart V4 in the ventral stream which is involved in processing shape and form information. We used Glass patterns as stimuli to investigate visual processing in both the dorsal and ventral streams, [36–38]. Glass patterns are constructed from random dot patterns composed paired dots (dipoles). By selectively specifying the relative orientation of the dipoles, the global form information–or global structure–changes (Fig 1). To perceive the global structure it is necessary to integrate the information conveyed by the local organization of the dot pairs over extended visual space. In the case where there is no global organization–a random dipole pattern–no structure is perceived (Fig 1C). Dynamic Glass patterns are constructed by presenting multiple images with the same global organization in a rapid sequence. Viewers perceive the stimulus as moving although with an unidentifiable motion direction [39,40]. The patterns elicit selective neural activity in both the dorsal and ventral streams [41], thus we aimed to activate both pathways and identify any differential development.

Using MVPA, we asked whether selective groups of voxels could distinguish differences between Glass pattern types as a function of area. Specific stimulus types were combined to form stimulus classes (e.g. dynamic stimuli) to produce a selection of contrasts (e.g. dynamic versus static). We collected data from seven individuals repeatedly across an age span of 3 months to 2 years of age. We found that classification performance gradually increased with age in all areas, and the ages at which significant classification emerged were surprisingly old.

## Methods

### Subjects

We collected data from seven non-human primates (*Macaca mulatta*, 2 female). Monkeys were supplied by Simian Laboratory Europe (Silabe, Strasbourg, France) and the German Primate Center (DPZ, Göttingen, Germany) and maintained in the animal housing facilities at the Max Planck Institute for Biological Cybernetics (MPI, Tübingen, Germany).

The study was performed in accordance with the German Animal Welfare Act (TierSchG) and Animal Welfare Laboratory Animal Ordinance (TierSchVersV). This is in full compliance with the guidelines of the Society for Neuroscience (SfN) and the EU Directive on the protection of animals used for scientific purposes (2010/63/EU, 86/609/EEC). Evaluation of research was performed by external audit by independent international reviewers. The study was reviewed by the ethics commission consisting of scientists, laypersons and animal protection agencies (§15 TierSchG) and approved by the state authority (Regierungspräsidium, Tübingen, Baden-Württemberg, Germany). Institutional (non-university) welfare reviews were done by the Max Planck Institute. Additionally compliance was ensured by regular inspections by state agencies (Regierungspräsidium), furthermore in-house welfare officials continuous monitored well-being of the animals.

Animals were held according to the latest insights regarding husbandry and (veterinary) care. Animals received ad-lib food and liquids and were kept in social groups of two or more. Social housing in large cages with environmental enrichment and natural lightning fostered natural behavior such as jumping, swinging, grooming and vocalizing. Environmental conditions such as temperature and humidity were optimized for the animals. Animal behavior was monitored continuously over closed circuit TV and at cage side by staff skilled in primate behavior. After the experiments the recovery from anesthesia was monitored and normal conditions were restored. The animals were not sacrificed after the longitudinal study.

From start to finish of the project the monkeys ranged in age from 102 to 1431 days (3.3 months to 3.9 years) at the time of scanning. At this age basic visual abilities correspond with 13 months and 16 years of age in humans. [7,42,43]. Four subjects were tested longitudinally (4 to 10 scan sessions roughly every other month), subject F08 was tested twice 2 weeks apart, and the two oldest subjects (B12 and C12) were each tested once. In total 30 individual data collection sessions were conducted over 9 years. Some data from two subjects (I03 and H03) have been published as part of an earlier study [30]. Here, we present data collected using a different stimulus type—Glass patterns rather than polar checkerboards—and an analysis not previously employed: multivariate instead of univariate techniques.

Subjects were scanned while anesthetized and paralyzed, in accordance with the protocols described in Logothetis et al. [44] and Kourtzi et al. [30]. Monkeys were premedicated with glycopyrrolate (0.01 mg/kg, i.m.) and ketamine (15 mg/kg, i.m.). After intubation and intravenous access, anesthesia was induced with a combination of an opiate (Fentanyl, 3 μg/kg), barbiturate (Sodium thiopental, 5 mg/kg), and a paralytic (Succinylcholine chloride, 3 mg/kg). Anesthesia was maintained by administration of remifentanil (0.5–2 μg/kg/min). To ensure acquisition of MR images that were free of movement artifacts, a muscle relaxant was administered (mivacurium chloride, 5 mg/kg/h). Fluids were continuously administered as lactated ringer with added glucose. Animals were ventilated (Servo Ventilator, Siemens, Germany) throughout the anesthesia period with the physiological parameters maintained as follows: pCO_2_(et) 30–35 mm Hg; spO_2_ 95–100%; temperature 38.3–39.3°C.

This standard protocol was adapted for infants as described in [30]. In infants, the initial paralytic Succinylcholine chloride was not administered and the dosage for remifentanil and mivacurium chloride were reduced to approximately 20% of the adult dosages.

### Imaging

For the MR images we used a vertical, 40cm bore, 4.7 Tesla, Bruker Biospin scanner. Echo planar images (epi) were acquired for measuring BOLD responses. Every brain volume was acquired in 8 k-space segments (8 shots), with a repetition time of TR = 6 s. The transversal slices were 2 mm thick and the voxel surface varied between sessions ranging from 0.5 to 0.75 mm^2^. Anatomical scans were acquired with FLASH and/or turbo spin echo (RARE) methods with a resolution ranging from 0.25x0.25x2.00 to 0.50x0.50x2.00 mm. fMRI images were corrected for motion artifacts (typically <1 mm), and any linear intensity trend was removed per slice and per voxel. 95% of the voxels had an intensity adjustment of 9% or less from mean intensity (Brainvoyager QX 2.3, Brain Innovation, Maastricht, The Netherlands, [45]). No spatial smoothing was applied. All subsequent analyzes were performed with Brainvoyager in combination with custom Matlab routines (Matlab 8.3, Mathworks, Natick, MA, USA)

### Stimuli

The visual stimuli were presented with a projector that extended the image into the magnet by using fiber-optic bundles for each eye (Silent Vision, Avotec, Stuart, FL, USA, see [30]). The monitor had a frame rate of 60 Hz and a resolution of 1024 by 768 pixels. The center of each of the two images was aligned with the fovea of each eye, using a fundus camera aligning the fiber bundle with the optical axis of the eye. Stimulation was binocular, except for one session (F09 15.5 mo) in which case we chose the best response for monocular stimulation. The viewing angle was 30 by 23 degrees horizontal and vertical.

The visual stimuli consisted of rotating checkerboards and Glass patterns of different configurations [26,36,38]. Fig 1A and 1B show examples of Glass patterns of concentric and radial type as they were presented to the subjects.

All structured Glass patterns were presented with 100% coherence. This means that all dipoles conformed to the orientation rule, either concentric or radial. The random dipole pattern comprises dot pairs that do not follow any orientation rule, resulting in random positions and orientations (Fig 1C).

In addition to using three different pattern types, the stimuli were presented either statically or dynamically. In the static version a pattern (e.g. radial) was presented for one second before it was refreshed and a similar newly-generated pattern (also radial) was presented. For dynamic Glass patterns, a new stimulus was presented every 33 ms. This gives an impression of a moving image without a discernable direction. Patterns had a dot density of 10 dots/deg^2^, dot size was 0.12 deg and the distance between the dots was *dx* = 0.414 deg.

To confirm general visual responsiveness for the fMRI BOLD activity at the beginning of each session, we used high contrast dynamic radial checkerboard, or “pinwheel”, stimuli. This stimulus resembles a dartboard with 15 deg wedges of high contrast black and white patterns in three concentric rings. The pattern rotated at 5 deg/s and changed from clockwise to counter-clockwise every 5 seconds. The pinwheel alternated with a blank background every 48 seconds. On average pinwheels were presented for 9.4 min (at least 3.2 min up to 28.8 min) per session.

The visual stimulation was aligned to maximized BOLD activation from these pinwheel stimuli before other measurements were taken. In four sessions (F09 at ages 11.3 mo and 21.5 mo, I03 at 4.8 mo, and M08 5.5 mo) the experiment was aborted because of lack of robust and significant BOLD activation in V1 even after realignment of the stimulus apparatus. The depth of anesthesia might have interfered with the BOLD activity in these sessions.

### Experimental design

Palindromic blocks of Glass patterns were presented for 36–48 seconds (i.e. stimulus types A, B, C were counter-balanced as: A-B-C-C-B-A). Blank screen epochs were presented for 24–36 seconds between every Glass pattern block. The total duration of Glass pattern presentation sequences ranged from 12.8 minutes to 1.6 hours (average 48.4 min) per session. Since runs started and ended with blank epochs the total duration of blank presentations was longer. Each complete scanning session lasted several hours. Note that small experimental details differed between sessions hence we provide the range for timing. However there are no systematic differences in the results across these minor differences, so data are combined across sessions and ages.

### Signal quality derived from a General Linear Model (GLM)

To determine the quality of BOLD recordings we used a GLM [46–48]. The model used (or “design matrix”) is the time course of the stimulus represented by a boxcar convolved with a double-gamma model of the Hemodynamic Response Function (HRF, [49]). The design matrix predicts elevated BOLD at a roughly 5 s delay after the stimulus was presented. To assess signal quality, GLMs were focused on V1 voxels only and not entire volumes. We examined both pinwheel and Glass pattern runs to confirm that the signal remains stable across runs. All types of Glass patterns were treated as equal (same predicted amplitude). A GLM model was fitted and amplitude and significance level (T-value) were calculated and examined.

In addition, a signal to noise ratio (SNR) was calculated. The amplitude of fundamental frequency of the stimulus alternation (*FF = 1/1*.*6 min*^*-1*^) was divided by the average of two flanking amplitudes at 0.5 *FF* and 1.5 *FF* (*f = 0*.*5/1*.*6* and *f ≈ 1*.*5/1*.*6 min*^*-1*^). These flanking frequencies are not higher harmonics of *FF*, therefor unrelated to the stimulus, and an estimate of signal independent noise. This approach was also used in our earlier study (see the figures 5A and 6A in [30]).

Both approaches revealed no systematic trend during the course of the experiment indicating stability of the signals. There was no systematic signal deterioration or improvement, so no additional exclusion of sessions was required.

### GLM maps of Glass patterns

To assess visual development Glass pattern responses were first analyzed with a standard GLM approach on all volumes including all voxels. In the GLM, the BOLD predictions for a Glass pattern and a blank are similar. This results in an *n*-by-*m* design matrix where *n* is the number of volumes (or time) and two (*m = 2*) number of predictors (blank or Glass patterns).

In determining the significance of BOLD activation a T-test was performed. For the significance threshold *p < 0*.*05* was set. This *p* value was corrected for multiple comparisons by expecting a False Discovery Rate of *q < 0*.*05* (FDR, [50]).

### Pattern analyses

In addition to GLMs we explored functional development in a more refined fashion and used multi-voxel pattern analyses on Glass pattern responses (MVPA, [31–34]) to characterize processing differences between different visual brain areas across different ages. A classifier was trained to distinguish between two classes of patterns. These classes were then used to *contrast* different features of the Glass patterns. We focused on three such comparisons. The first classification was ‘stimulus versus blank’. Here responses to all Glass pattern types were compared to responses during blank epochs with the purpose of characterizing basic visual responsiveness. In ‘dynamic versus static’, BOLD signals obtained during presentation of static Glass patterns were classified in comparison to signals evoked during presentation of dynamic patterns (including both structured and random). In ‘structured versus random’, signals obtained during static random dipole presentations are classified from those obtained from static Glass patterns that have a structured form (including both radial and concentric). In analyzing the BOLD response, corresponding time epochs are pulled from matching patterns. In most sessions all pattern types were presented (concentric, radial, and random dipoles), except for sessions of H03 and I03 where no radial Glass patterns were included. Sessions for subject F08 did not include random dipoles. In these cases we classified contrasts with fewer pattern types or when a contrast could not be constructed we dropped the session from the analyses for that particular contrast.

#### Feature selection

**Fig 2:**
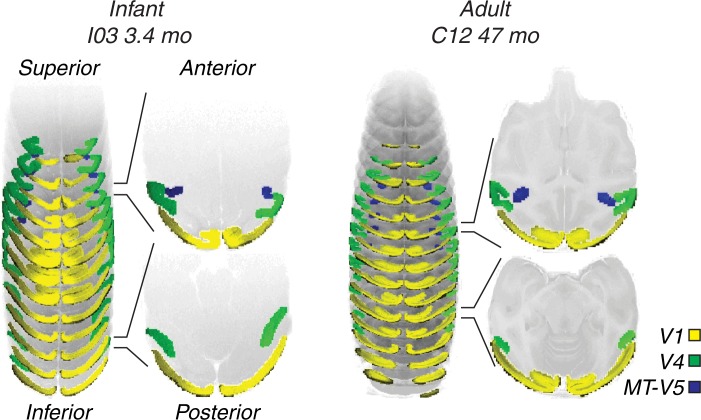
Example of volume selection. Left is infant I03 at 3.4 months of age; right is adult C12. The three target brain areas are color-coded (V1: yellow, MT-V5: blue and V4: green). Brain areas typically span multiple slices. Right of the stacked slices are two example slices with cross-sections.

The use of functional localizers to identify different brain areas is common in human fMRI studies, however we had no stimulus that is proven to drive BOLD in pre­specified areas in the infant brain. Localizers tap into the presumed function of brain areas (e.g. motion encoding), but infant brain areas might have overlapping (or multimodal) functions (see [51,52]). This means a hypothetical localizer would need to tap a variety of functions that are not known *a priori*. Using the signals from our “pinwheel” stimuli would have risked omitting responsive voxels if signals were weak or inconsistent. Therefore we preferred to use anatomical landmarks over the use of functional localizers, because these landmarks remain relatively consistent throughout development, e.g. basic visual area structure is established near birth and maintained throughout life in nonhuman primates [53,54]. Anatomical images were acquired at a higher resolution than functional data. These high-resolution images were used to identify the different brain regions. Guided by the Saleem and Logothetis [55] brain atlas, three brain areas were outlined in each anatomical slice. Fig 2 exemplifies this for the youngest and oldest sessions. The resolution of the anatomical slices was then resampled to match the functional ones. By stacking the 2 mm thick marked regions (regions of interest, ROIs) a 3D model of different brain regions was constructed. The three different volumes of interest (VOIs) were primary visual cortex (V1), and extrastriate areas V4 and MT-V5.

The voxels within each brain area were sorted such that the best responding voxels (highest T value) were used early in the classification analyses and the least responding voxels were used last. The rank-ordering within a VOI was done based on responsiveness to the pinwheel stimulus. Responsiveness was determined by whole volume T value maps. By using pinwheel activation, our metric was therefore independent from Glass patterns responses and the MVPA contrasts. We included all voxels that were positively responsive to visual stimuli.

The voxel ranking could have continued until all voxels of the VOI were included, but this would have resulted in a larger number of voxels for larger VOIs, making it unsuitable for fair comparisons between sessions. In addition, the number of voxels in large VOIs could outnumber the number of patterns. Depending on the scan duration and thus the available time to repeat Glass pattern sequences, the number of patterns varied from 32 to 189 (average 92.3).

Given the intrinsic noise of the fMRI signal, a large number of voxels could show a spurious correlation, which could potentially cause a false accuracy value that is either significantly higher or lower than chance. However, this signal would not persist but would appear as a chaotic oscillation. Therefore we chose a cut-off of the smallest possible number of voxels to retain equal-sized voxel pools across all VOIs. For all classifications, rank ordering was truncated at 70 voxels. For detailed discussion see [34].

Because the GLM results in V1 in response to pinwheel stimuli across all ages exhibited positive BOLD, we did not include negative BOLD in our analyses.

#### Pattern assembly

During functional imaging sessions different Glass pattern sequences were presented for up to 1.6 hours. In the MVPA, the BOLD signal is convolved with a boxcar function delayed by one time epoch–the time required to scan one volume, which corresponds to 6 s. These values are averaged over runs resulting in an *m*-by-*n* matrix, where *m* is the number of volumes and *n* the number of voxels. Subsequently the contrasts are prepared for the classifier training by combining the average BOLD responses of volumes within the same class.

#### Classifier training

We trained a support vector machine (SVM) linear classifier (SVMlite, [56]) to test for discrimination of stimulus patterns. The classifier was trained with a subset of data leaving out 1/8 of the condition pairs. To test for generalization, all our results were cross-validated with unused data. This sequence of training and testing was repeated 8 times. These 8 resulting accuracies were then averaged and the variance provides an estimate of the robustness of the mean.

Chance levels were determined by 95% probability of a binomial distribution with the number of trials being equal to the number of patterns used in the classifier training (*p*_*0*.*95*,*n*_). Classification performance sometimes drops below the lower boundary of the chance level. In these rare cases the classifier mislabeled a considerable portion of the data, a limitation that is often attributed to limited number of trials. These cases were also considered to be non-significant.

Both the classification variance and the chance level were used to determine the sensitivity index (*d’*):

$$d\prime =\frac{\mu_{acc}-\mu_{chance}}{\sqrt{\frac{1}{2}(\sigma_{acc}^{2}+\sigma_{chance}^{2})}}=\frac{\mu_{acc}-0.5}{\sqrt{\frac{1}{2}(\sigma_{acc}^{2}+(p_{0.95,n}-0.5{)}^{2})}}$$ (Eq 1)

with *μ*_*acc*_ and *σ*_*acc*_ being the average and standard deviation of 8 cross validations, and *p*_*0*.*95*,*n*_ the chance level expected by the binomial distribution (see above).

In order to determine the robustness of *d’*, data were resampled to 1000 bootstrapped datasets. From the 8 classification accuracies (computed as described above), 8 x 1000 accuracies were drawn with replacement. For each value of accuracy obtained, a new *d’* estimate was determined (Eq 1), yielding an average *d’* close to the non-bootstrapped mean and a variance reflecting the variance in the data. The resulting average *d’* values for each age were fit with the Naka-Rushton saturation function as per Eq 2:

$$d\prime (A)=d\prime_{0}+\frac{d\prime_{max}\cdot A^{n}}{A_{halfmax}^{n}+A^{n}}$$ (Eq 2)

where *d’*_*0*_ is a fixed offset, *d’*_*max*_ is the asymptotic level, *A*_*halfmax*_ is the age at half *d’*_*max*_, and *n* is a shape parameter that determines the slope. The fit minimizes squared error and uses trust-region optimization (Matlab 8.3, Mathworks). The offset is restricted to *d’*_*0*_ *= 0*. The asymptote (*d’*_*max*_) is restricted between *d’ = 0* and the maximal *d’* that can be achieved when the classifier uses the training set as a test set (i.e. the accuracy is close to 100%, *d’ ≈ 12*). The shape parameter *n* is kept between 0 and 4 to avoid fit results that approximate step-functions. The parameter *A*_*halfmax*_ is not restricted.

To define a significance age, we chose the conservative criterion of the age at which classification accuracy increases above *d’ = 2*. This value corresponds roughly to one standard deviation above chance (*d’ = 1*.*93*), meaning *d’ = 2* is slightly more restrictive. In determining whether *d’* values of conditions or VOIs are statistically different, the 95% confidence intervals were used from the bootstrapped datasets.

## Results

**Fig 3:**
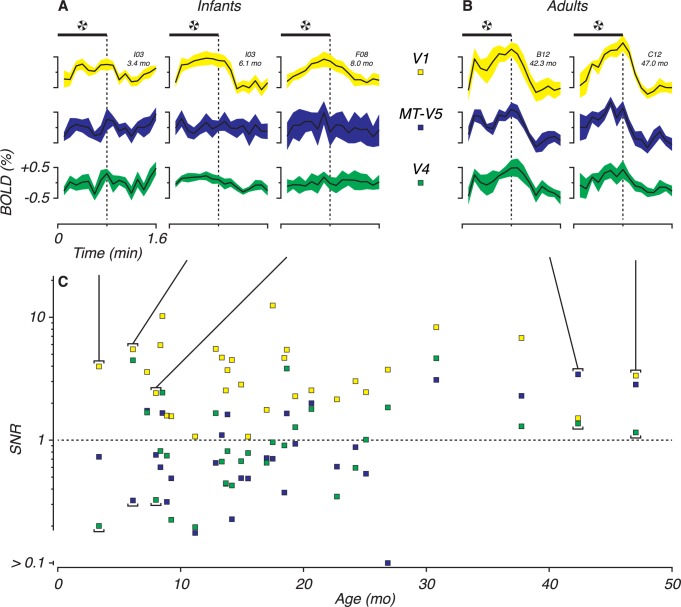
Response to pinwheel stimuli. Voxels in areas V1 (yellow), MT-V5 (blue) and V4 (green). (A,B) Average BOLD for five representative sessions, three from infants (left, panel A) and two from adult (right, panel B). Solid black lines are average BOLD activation to pinwheel on-off alternations (8–12 repetitions). colored areas are standard deviations. The horizontal black bars indicate the time of stimulus presentation. (C) Signal divided by noise (Signal to noise ratio, SNR) as function of age. Signal is the amplitude at the frequency of the stimulus alternation (*FF*). The noise is the average of the amplitudes of the flanking, not stimulus related frequencies (0.5 *FF* and 1.5 *FF*). The signal exceeds noise when *SNR > 1* indicated with a dashed line.

We scanned 7 monkeys over an age range of 3 months to 4 years of age and analyzed a total of 30 fMRI sessions. Across all ages we observed significant BOLD activation in V1 voxels (Fig 3, yellow). The SNR in extrastriate areas (V4 and MT-V5) remained low for sessions at young ages and showed a delayed development. This is in accordance with what we reported earlier [30].

In the current study we also measured changes in BOLD activation in response to presentations of globally different *Glass patterns* in three different brain areas: V1, MT-V5 and V4. Responsiveness to Glass patterns increased with age in all three brain areas.

### Generalized linear model

**Fig 4:**
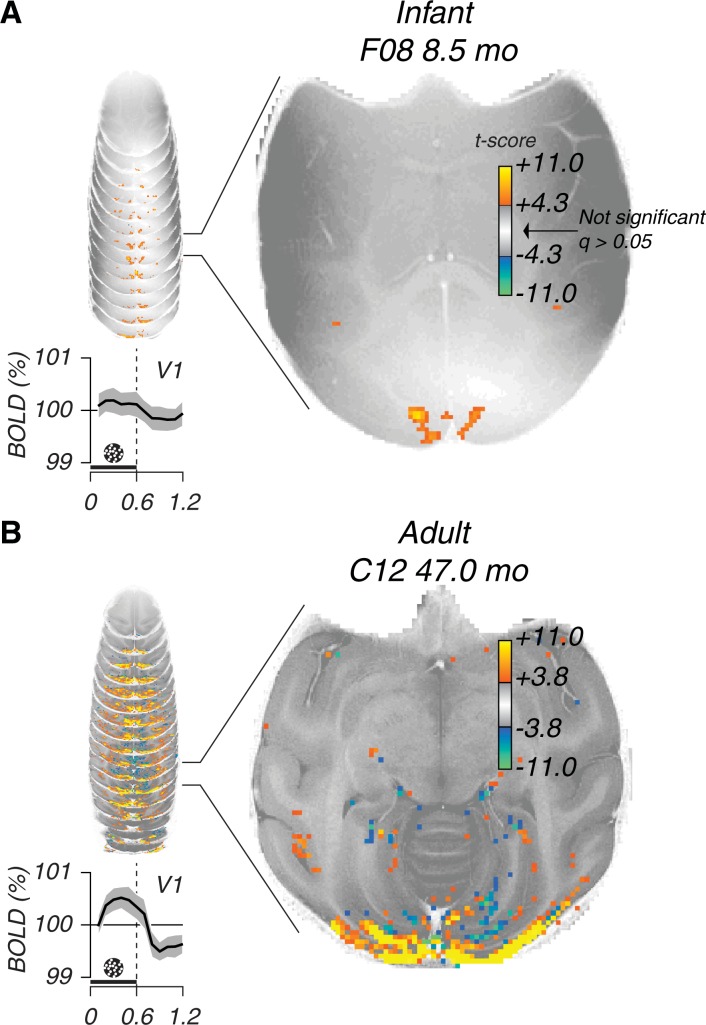
Two examples of generalized linear model maps. (A) a session from a young subject and (B) a session from an older subject. The stacked images on the left are anatomical slices with GLM maps superimposed. One slice is exemplified next to the stack of slices. The GLM color maps are T-values (t-test) thresholded at a significance value that is corrected for false discovery rate (FDR, *q < 0*.*05*, see Methods). Traces below the stacked images are average BOLD response to Glass patterns in V1. Layout is similar as Fig 3 where pinwheel responses are shown.

BOLD signals were first analyzed with a standard generalized linear model (GLM) approach. Fig 4 highlights voxels on an anatomical background that have significantly different levels of BOLD between blank and all Glass patterns combined (t-test, *p < 0*.*05*, corrected with *q < 0*.*05*, see Methods). For the GLM maps all Glass pattern types were pooled together. The top panels (Fig 4A) show a session at a young age (8.5 mo) and the lower panels (Fig 4B) show an older age session (4 years). From the stack of slices on the left in Fig 4 a single example slice is extracted and shown on the right. Below the stacked slices is the average BOLD response in V1 to Glass patterns. This activation pattern is comparable to the pinwheel responses (Fig 3). When comparing the insets the peak-to-peak modulation is noticeably larger in the adult session.

Only minor activation was observed in anterior areas of both hemispheres in the infant session illustrated in Fig 4A. Activation in the superior temporal sulcus (STS) is marginal in infants. Posterior activation clusters medially in the calcarine sulcus, which indicates activation by the peripheral visual field. In contrast a group of voxels (a cluster) is clearly active in the adult session. These activation patterns elicited by Glass pattern stimuli are consistent with our earlier results obtained with pinwheel stimuli [30].

**Fig 5:**
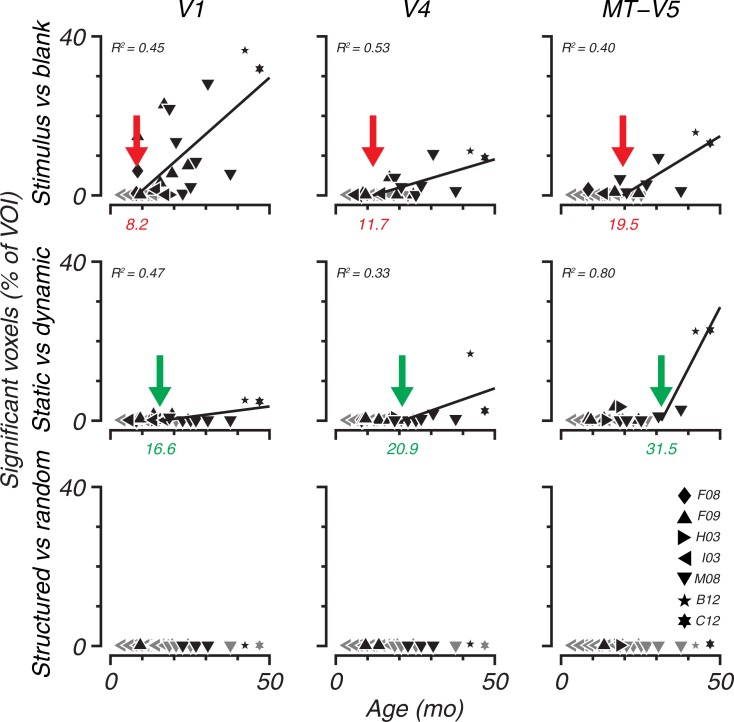
Percentage of voxels from total VOI that significantly modulated. (GLM, t-test *p < 0*.*05*, Bonferroni corrected for number of voxels within VOI) as response to Glass patterns (GPs) versus blank, Dynamic GPs versus Static GPs, and Structured GPs versus Random dipoles. Gray symbols indicate sessions where no voxels were significant. Symbol types indicate different subjects. All significant sessions are used to fit a line when four or more consecutive sessions have active voxels (linear regression by minimizing squared errors) and the explained variance in the subset of sessions are indicated as *R*^*2*^ (Note that all sessions are considered to be independent and colinearity or covariances are ignored). The intercepts of the lines with the abscissas are indicated with colored arrows. The ages at this intercept are shown below the axis. These values reflect the ages at which significant voxels start to appear in the VOIs. In the lowest panel (Structured versus Random) an intercept between 0 and 50 months could not be obtained.

Fig 5 shows the percentage of voxels within each marked volume that significantly modulated based on the GLM. Contrasts are shown in rows and VOIs are shown in columns. The black symbols indicate sessions with significant activation; different symbols indicate different animals. The colored triangles indicate the age at which significant BOLD modulation is reliably evident for each of the contrasts indicated. Our criterion for reliability was at least four consecutive sessions with significant voxels; the lines are regression fits to the data from the first of four to all subsequent significant sessions in each panel. In general, for stimulus vs. blank (top row), there is clear activation in all three visual areas, with V1 showing substantially earlier significant activation (9 mo) than other areas. For the contrast static versus dynamic stimuli (middle row), all three areas show reliable BOLD modulation only after 20 months in the extrastriate areas. MT-V5 showed delayed onset compared to V4 by this analysis. Although there was variation across sessions, with some sessions showing only small amounts of activation even in older animals, the developmental trend is clear. Interestingly, none of the three cortical areas showed reliable BOLD modulation to the structured versus random contrast (bottom row), suggesting that the underlying activity reflected primarily local stimulus structure.

All Glass pattern types were pooled together for the analysis shown in Fig 5. The percentage of significant voxels is greatly reduced for analysis of individual Glass pattern types (i.e. not combining across all Glass patterns). It may be that the GLM method is not sensitive to subtle differences in local activation that may accompany the different Glass pattern types since it is based on *single* voxel comparisons of BOLD in individual subjects.

### Multivoxel pattern analyses

GLM analyses are designed to do voxel by voxel comparisons, thereby ignoring the reality that neighboring voxels belonging to the same cortical area may perform similar processes. Therefore activation arising from *multiple* voxels (or pools) could remain subthreshold in GLM, but might surface in MVPA. MVP analyses compare activation patterns on the pool *within* a specific session and thus in our case *within* age.

We tested voxels from the same three visual areas, V1, V4, and MT-V5, with MVPA. The trained classifiers were each tested on three different contrasts. The contrast ‘stimulus versus blank’ has a strong univariate response across multiple voxels, which is reflected in the results of the GLM analyses. We repeated this contrast for the MVP analyses to compare accuracy with other contrasts.

**Fig 6:**
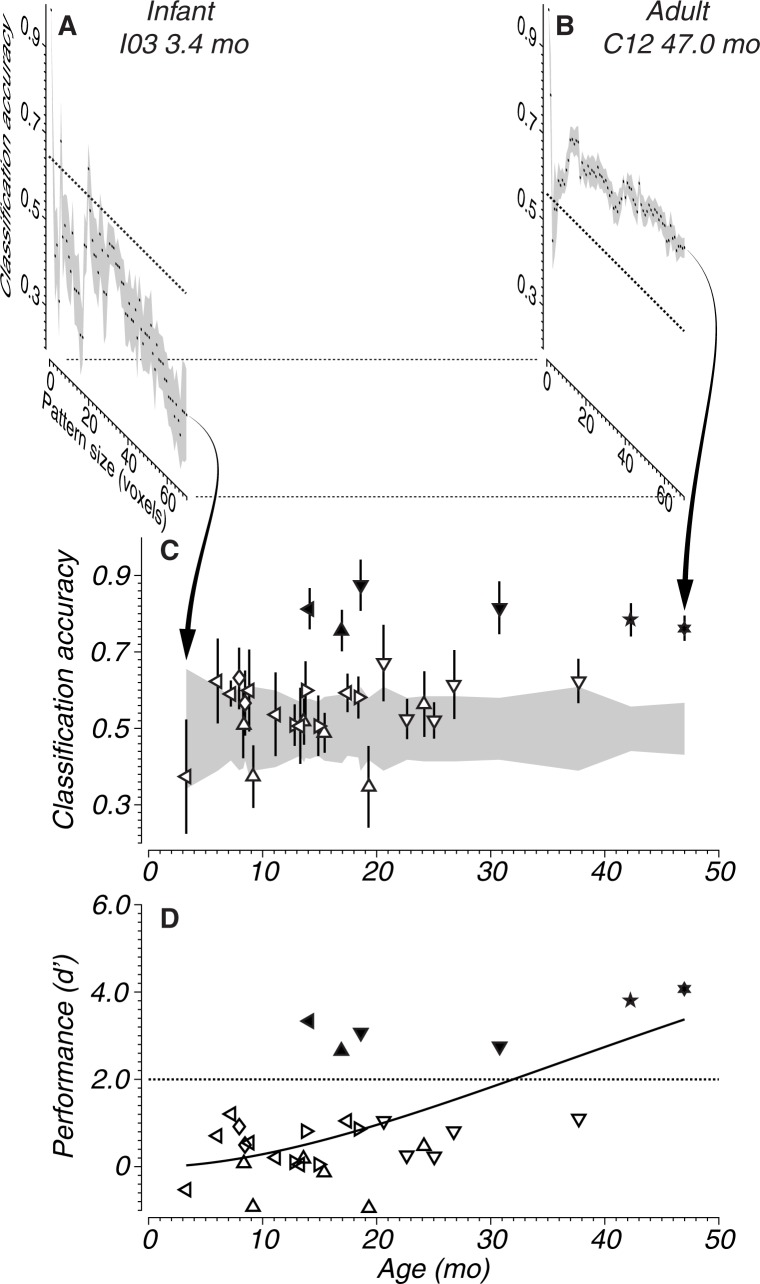
Results of MVPA classification of MT-V5 voxels contrasting stimulus against blank conditions. (A,B) Classification accuracy as a function of pattern size for two subjects, one young and one adult. Squares indicate a mean of 8 cross validations. The gray shaded area is the standard error of the mean. The dotted lines are the chance threshold expected from a binomial distribution. (A) Subject I03 at 3.4 mo. (B) Same contrast shown for an adult (Subject C12 at 47 mo). (C) Classification accuracy as function of age for all subjects and sessions. The shaded area reflects chance threshold as determined by a binomial distribution. Open symbols indicate accuracies lower than threshold. Vertical lines indicate standard deviation. Each subject is indicated with a different symbol type (see lower-right panel in Fig 5 for identification). Arrows point to the classification results exemplified in the top panels A and B. (D) Transformation of classification accuracies to sensitivity index *d’* (see Methods). *d’* is plotted as a function of age for all subjects. The dotted line represents chance; solid and open symbols represent above chance and non-significant classifications, respectively. The solid line represents a fitted function (Naka-Rushton, Eq 2) capturing the relationship between *d’* and age.

Fig 6 shows accuracy traces of the youngest (Fig 6A) and oldest (Fig 6B) session for the contrast ‘stimulus versus blank’ in area MT-V5. The horizontal axis depicts the number of iterations with which the classifier was trained and tested. At every new iteration a voxel is added (with the best voxels added first) and the pool is enlarged. With larger pool size a more robust classification between the conditions in the contrast can be expected. If the activation patterns were different between the conditions, it would give rise to higher accuracies as shown on the vertical axis. As the pools are made larger the accuracy traces level off, the added voxels do not contribute to higher accuracies (see [34]).

An accuracy trace from the first session of the youngest animal (I03, age 3.4 mo; Fig 6A) shows that, even after adding 70 MT-V5 voxels to the pool, classification accuracy does not rise above chance. In contrast, the accuracy of the adult example (Fig 6B, C12, age 47 mo) rises above chance quickly and saturates by around 15 voxels. In both illustrated sessions, accuracy is unstable when patterns are smaller than 10 voxels. With a small number of voxels the SVM achieves very high (100%) or very low (0%) accuracies. This is a stochastic process caused by a small number of voxels.

In the following analyses, we used the accuracy values of the 70th voxel as the metric for classification performance. Fig 6C summarizes the accuracies for all subjects and ages where MT-V5 voxels are used to classify ‘stimulus versus blank’. As a result of different experimental trial lengths and number of stimulus repetitions across sessions (see Methods) the chance level varies across sessions. This is represented by the gray shaded area around an accuracy of 0.5 in Fig 6C. To take account of this variation of chance level, the accuracy data were transformed into a sensitivity index (*d’*). *d’* as a function of age is plotted in Fig 6D. We adopted the criterion of *d’ > 2* for significant classification (see Methods section Classifier training). As of age ~12 months some sessions show above chance accuracy, which are indicated with filled symbols.

**Fig 7:**
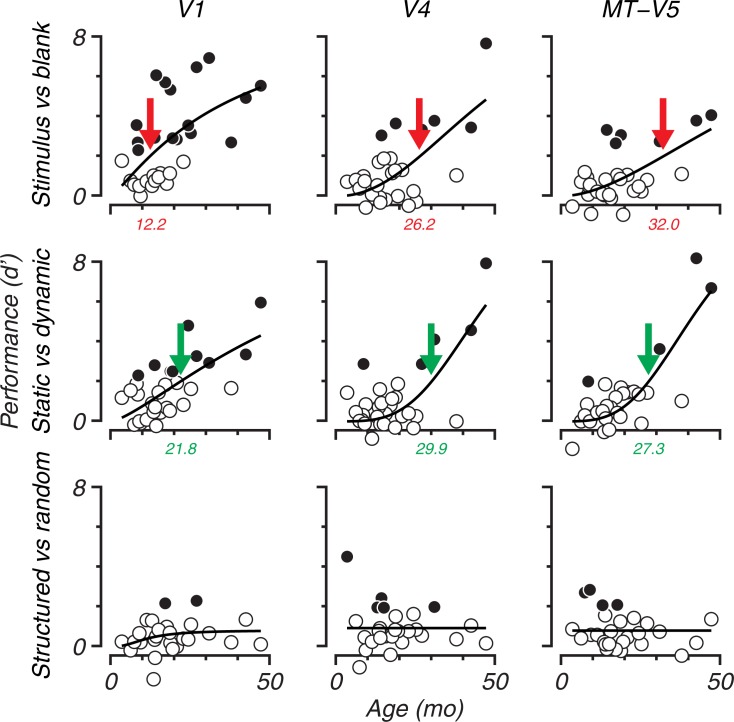
Development of *d’* as function of age for all contrasts and areas. Sensitivity indices are presented in the same 3 by 3 grid as in Fig 5: Contrasts are represented in rows and brain areas are represented in columns. Note that data presented in Fig 6D appear in the upper-right panel. Open symbols indicate sessions with below chance accuracies, meaning that the average accuracy (including one standard deviation) did not exceed the chance level as determined by a binomial distribution (*p*_*0*.*95*,*n*_). The colored arrows show the age when the fitted function (Naka-Rushton, Eq 2) exceeds *d’ > 2*. In the upper-right panel this corresponds to the intersection of the dotted and solid lines from Fig 6D at 32.0 mo.

Where Fig 6 only shows one contrast (stimulus vs. blank) for one cortical area (MT-V5), Fig 7 shows sensitivity indices of all brain areas and contrasts used in the MVPA. As in Fig 5, contrasts are shown in rows and cortical areas (VOIs) are shown in columns. In general, classification accuracies increase with age. However, the age at which classification accuracy is consistently higher than chance varies with contrast and brain area.

As noted above, to quantify the ages when accuracy is reliably above chance we took the age when the sensitivity index, *d’*, exceeds 2. These ages are indicated by colored arrows in Fig 7.

Area V1 reliably showed the earliest classification of the three areas tested. The contrast ‘stimulus versus blank’ (top row) yielded above chance accuracy at the youngest age in V1 (Fig 7, red arrow, 12.2 mo). Sampled from 8 cross validation results the bootstrapped 95% confidence interval (see Methods section Classifier training) is between 11.7 and 13.2 months (Table 1). The classification remained above chance beyond 12.2 months. Significant accuracies in extrastriate areas V4 and MT-V5 appeared later, around age 2 years. For V4 *d' = 2* at 26.2 mo and for MT-V5 32.0 mo. None of the bootstrapped values were smaller than those of V1 (*p < 0*.*001*, Table 2A).

**Table 1:** Intersecting age in months at *d’ = 2*.

	V1	V4	MT-V5
Stimulus vs. blank	12.2 (11.7–13.2)	26.2 (22.9–27.5)	32.0 (26.2–35.0)
Static vs. dynamic	21.8 (18.6–22.6)	29.9 (26.0–31.3)	27.3 (23.7–28.7)
Structured vs. random	all remain below *d’ = 2*		

Values between brackets are 95% confidence intervals.

**Table 2:** Significance values (*p*) for difference in classification ages.

A: Stimulus vs. blank				B: Static vs. dynamic			
	V1	V4	MT-V5		V1	V4	MT-V5
V1		<0.001	<0.001	V1		<0.001	<0.001
V4			0.006	V4			0.879

Significance for classification ages that are older in areas in rows than in columns.

For the ‘static vs. dynamic’ contrast (Fig 7 and Table 1, middle row), V1 also classified earlier than the extrastriate areas (21.8 mo, V1 earlier than V4: *p < 0*.*001*, V1 < MT-V5: *p < 0*.*001*, Table 2B). However the age of significant classification for this contrast in V1 appeared later than for ‘stimulus versus blank’ (Table 1 and Fig 7 left, compare green and red arrows, *p < 0*.*001*, Table 3). Also in V4 classification is later for ‘static vs. dynamic’ (*p = 0*.*006*). The earlier classification of ‘static vs. dynamic’ in MT-V5 is not statistically different (*p = 0*.*902*, Table 3 and CI overlap in Table 1). In extrastriate areas, accurate classification appeared at roughly 25–35 months for both areas and seemed to develop similarly for ‘stimulus vs. blank’.

**Table 3:** Significance values (*p*) for classification age in stimulus vs. blank being older than static vs. dynamic.

V1	V4	MT-V5
<0.001	0.006	0.902

Surprisingly, form classification (‘structured versus random’) did not reliably exceed *d’ = 2* (Fig 7 and Table 1 bottom row). Although in some sessions the sensitivity index indicated significant classification accuracies, no clear developmental trend was evident. For this contrast, V1’s classification performance is as poor as that in the extrastriate areas. The general trend of V1 showing reliable significant BOLD signals earlier than extrastriate areas is clear for the first two contrasts. V4 and MT-V5 do not significantly differ from each other with respect to classification age regardless of the contrast.

## Discussion

We conducted a developmental study of functional MRI in infant monkeys ranging in age from 3 months to 2 years. Our goal was to chart the relative development of coherent activation of dorsal and ventral stream areas using Glass pattern stimuli, compared to V1, the primary visual cortical area. We used two analysis approaches: GLM and MVPA, to identify the onset of reliable BOLD activation of V1, V4, and MT-V5. Both analyses showed later coherent activation of extrastriate areas compared to striate cortex, but V4 and MT-V5 were not consistently different in the age at which coherent activation was reliably present. Of the three contrasts we used, stimulus presence/absence was the most robust with ‘dynamic versus static’ revealing reliable but later activation in some cases. None of the visual areas we tested yielded reliable results based on the structure of static Glass patterns. Even with more sensitive MVP analysis we did not find significant classification of activation patterns earlier than GLM. However, in all cases, the ages of reliable classification were much older than would be expected based on neurophysiological investigation of the same visual areas in monkeys (cf. [9,10]). These classification ages also do not correlate with the onset age of basic visual processing since behavioral ability to perform related perceptual discrimination tasks is apparent much earlier than the BOLD activation would suggest [26]. However, note that these observations are specific to *Glass pattern* activation. *Pinwheel* activation is observable across all ages and BOLD activation is already robust early on (see Fig 3). The Glass pattern activity reflected by the fMRI may instead be related to maturation of higher-order organization of visual function.

### Comparison to previous studies in human infants

When comparing results from human infants–taking account of relative developmental pace, equating weeks in monkey to months in human [43]–EEG results show sensitivity earlier than we found accurate classification for Glass pattern types at equivalent monkey age of 4–5 weeks [57]. Also, our results seem to contradict fMRI results of Biagi et al. [58]. They observed activation in young human infants (~7 weeks, equivalent to ~2 wo monkeys) when contrasting coherent with random moving dot fields. They report that only the extrastriate visual cortex exhibits increased BOLD, not V1. Adult V1 however shows *reduced* activation for coherent motion compared to random motion. They suggest the development of V1 is delayed with respect to extrastriate cortices and areas like MT-V5 are driven by a parallel pathway and thus bypassing V1. When contrasting motion versus blank the results for the extrastriate visual cortex are somewhat heterogeneous. About half of their tested infants show decreased BOLD where the others show positive BOLD. The polarity also seemed to switch within subjects across hemispheres.

There are some substantial differences between the studies that make a direct comparison difficult. Importantly, their stimuli evoke a strong percept of coherent motion, at least in adults, and strongly drive neurons in macaque area MT [59] as well as neural activity in humans ([60], albeit stronger in V1 [61]). Such robust single cell responses have not been demonstrated for coherent Glass patterns, but BOLD activation has been documented in human adults [33,41]. Additionally, in our approach, we used VOIs defined by morphological waypoints. Biagi and colleagues used an iterative correlation technique to select visually responsive voxels. Our more conservative approach treated voxels within a VOI equally. Only when performing MVPA classification did we rank-order according to pinwheel activation.

Yamada, Morita and their colleagues [62,63] report in two studies that when data from several anesthetized human infants are pooled, they exhibit stimulus-related BOLD in V1, in contrast to Biagi et al. [58]. In a pool with gestation-corrected age ranging from 0–5 wks [62] or 4.4–8.4 wks [63] BOLD was positive. Negative BOLD was observed in a pool with older infants (≥8 wks, [62]; ≥13.4 wks, [63]). Altman and Bernal [64] reported that negative bold can be observed in occipital areas of 3 mo to 9 yo anesthetized human infants and children. Awake teenagers (10–14 yo) only showed positive BOLD. In all three studies a flickering light was used, bright enough to visually stimulate with the eyes closed. In our youngest subjects, and without grouping subjects, we also observed V1 BOLD in response to checkerboard stimulation. However with a far more subtle stimulus (Glass patterns) we only observed BOLD at later ages.

Recently Deen et al. [65] showed that awake human infants (range 3–8 mo) exhibit a differential response to visual categories that cannot be explained by lower level feature differences. Spatial organization of BOLD activation was remarkably adult-like. Their full-color human infant-oriented movies were well-suited to the infant subjects. Our stimuli were not specifically tuned for infant selectivity given the aim to make developmental comparison with adult selectivity. Deen et al. used innovative but unconventional techniques to eliminate noise-prone data segments and reduced their dataset to 18% of its original size. Our approach was different; we included as much data as possible. By using anesthetized subjects we had the advantage to, a priori, reduce a major source of noise: motion artifacts.

Across these studies, differences in methodological preferences make it hard to compare results. However, all cases except Biagi et al. [58] are consistent with our data showing early activation in V1 to various visual stimuli.

### GLM and MVPA

We previously applied GLM analysis to developmental fMRI activity evoked by large-field rotating checkerboard stimuli [30]. Our current GLM results of Glass pattern activation are consistent with our previous study in that significant onset of V1 activation precedes downstream extrastriate areas. In both cases, coherent V1 activity is detectable before 12 months of age while activity in extrastriate areas becomes reliably present thereafter. However, activity evoked by high-contrast checkerboard stimulation was generally evident in all areas earlier than that evoked by the Glass patterns. This is perhaps not unexpected since Glass patterns are less salient stimuli than rotating checkerboards. In particular, the dipole patterns that are the basis for Glass patterns are not especially strong stimuli for V1 and V2 [38,66]. While the local coherent orientation information in linear Glass patterns is sufficient to drive orientation selective neurons, the global organization in Glass patterns is not reflected in neuronal responses in these early visual areas. Although there are no published neurophysiological data on Glass pattern responses from downstream visual areas, fMRI studies in human adults suggest that these areas can signal the global form in Glass patterns [33,41,67]. Nevertheless, we were able to identify coherent responses from all three visual areas to Glass pattern stimulation, although later than with checkerboards. It is both interesting and important that the apparent development of reliable BOLD activation is stimulus dependent.

Our aim in employing MVPA in comparison to standard GLM analysis was to identify any subtle developmental trends that were not detected by the GLM approach. Remarkably the two approaches yielded similar results for the contrast ‘stimulus versus blank’, which would be expected given the strong univariate signal in that comparison. However, contrasting dynamic versus static and structured versus random Glass pattern classes also yielded generally comparable ages for MVPA and GLM, which can not be explained by a general (univariate) signal increase and reflects a functional distinction.

With both methods we identified different classification ages for processing in V1 and extrastriate VOIs (Fig 7). V1 shows reliable activation by age 2 years and extrastriate areas are relatively delayed. However, the GLM analysis yielded an *earlier* significant developmental trend in V4 than MT-V5 for this contrast, while the classification analysis converged on *similar* ages for V4 and MT-V5.

In contrast to our expectation, the GLM analysis generally established earlier significant age points than MVPA, at least for ‘stimulus vs blank’, which contradicts the notion that the MVPA classification approach is the more sensitive one. When overall signal strength is low, even MVPA may perform poorly. For instance, the oldest session of subject M08 (~38 mo) showed a substantial drop in signal strength from earlier sessions, which was independent of the analysis approach. There were no other indicators that anything was unusual in this session; the same inclusion criteria were satisfied and a response to rotating checkerboard stimulation was observable. It is difficult to identify the source of variation in the BOLD response within a particular session. There may be subtle changes in physiological parameters that result in a reduction in signal to noise ratio over time. Whatever the case, the general convergence of the results from the two methods strengthen the impression of a relatively late development of coherent BOLD activity in downstream areas in infant macaques under our imaging conditions.

Some limitations on the outcome of our study may be related to choices of stimulus and analysis parameters. We chose to use Glass patterns since they have previously been shown to activate both dorsal and ventral stream areas [41]. However, if these areas are most responsive to the local dipole patterns rather than the global structure, then they will respond similarly to coherent and random dipole patterns. In that case, it would not be surprising that we found no reliable selectivity for the contrast ‘structure versus random’ and hence no developmental trend. Another consideration is that the ‘blank’ stimulus is not equivalent to ‘no stimulus’ (black). In between Glass pattern presentations, a gray, 30 deg field of the same mean luminance was presented in an otherwise dark scanner environment. We did this to eliminate the potential confound of a luminance response and to focus on functional differences between stimuli. This means that the ‘blank’ signal potentially evoked a response as well, and given that luminance was matched to the extent possible, the signal evoked by ‘stimulus’ might have been comparable to the ‘blank’ stimulus. Finally, given the restricted time available for each scan session, we opted to use fixed stimulus parameters (e.g., dot size, density, speed) and vary only the form (concentric, radial, random; static, dynamic). These parameters were chosen to be perceptually salient at all ages based on previous psychophysical data [26,68], however, they may not have been equally well matched to the underlying neural populations at each age.

In terms of the analysis approaches, the GLM methods we used were quite standard. For the MVPA, it may be important to note that we chose to use a fixed number of significant voxels across all three brain areas and across ages. We did this to avoid any confound due to the overall size of the VOIs across age and animals, and to avoid bias due to different overall levels of activity across VOIs. Since we used both GLM and MVPA, the fact that the results are similar across methods suggests that particular choices with respect to the analysis parameters are unlikely to have caused substantial limitations on the results.

Also with respect to the MVPA, when correlating stimulus and response we used a conservative 6-second delay. Little is known about hemodynamic response (HRF) development in monkeys. Harris et al. [69] identified several reasons for hemodynamic coupling to vary during development. In addition, Arichi et al. [70] reported a shift in delay from ~11 seconds to ~5 seconds in humans around birth. For our analysis, we kept the HRF delay the same for all sessions to maintain consistency across ages. However, we did examine the phase shift of the 10% highest amplitudes in V1 voxels at the checkerboard stimulation frequency. In 27 of 30 sessions the phase deviation was less than 6 seconds, which corresponds to a shift within the sampling time of a single volume. The 3 sessions with larger deviations were not confined to a particular age group and belong to sessions with low SNR (Fig 3) that consequently made the phase estimate uncertain. We think it is unlikely that our conservative HRF delay was a limiting factor for classification accuracy. We did not find a systematic shift in our infant monkeys as was observed by Arichi and colleagues in newborn humans.

The sign of the BOLD response may differ between infants and adults. Some studies report that negative BOLD is a better indicator of neuronal signals in infants [64,71–73], while in adults, negative BOLD is suggested to play a role in larger network activity (default-mode network: [74]). We observed less negative BOLD in area V1 than in more anterior areas. Also, some sessions had a substantial number of negative BOLD voxels but these sessions were not peculiar to infant monkeys, were not more prevalent at any particular age, and did not correlate between VOIs within the same session. We observed that the sessions with substantial negative BOLD voxels also had low T-values, indicating the voxel selection was performed closer to zero where sign switching is more common, thus yielding a larger proportion of negative BOLD voxels. Since there was no consistent pattern to the prevalence of negative BOLD, particularly in the infants, we did not include it in the analyses.

### Anesthesia and alertness

Martin and colleagues reported that negative BOLD to some degree depends on the anesthesia protocol used [71]. Their pattern of BOLD is similar to ours, but they also found that the number of subjects exhibiting negative BOLD was different for different anesthetic agents. In addition, Biagi et al. [58] reported no negative BOLD in awake human infants, which they relate to the state of alertness. The fact that negative BOLD seems to depend on the anesthesia protocol or attentiveness raises even more caution for assuming any consistent relationship to development.

Of course, anesthesia affects sensory processing. In mid-level visual processing, the local cue integration abilities of MT neurons are reduced [75]. Because neuronal processing of stimuli that require figure-ground segregation is disrupted in V1 neurons under anesthesia [76], it is suggested anesthesia has an effect on cortical feedback. Possibly temporal network dynamics are affected. Multi unit activity (MUA) and local field potential (LFP) activity are reported to be dissimilar under anesthesia compared to awake [77].

The physiology studies mentioned above used isoflurane as a main anesthetic whereas we used the opiate remifentanyl [44]. The type of anesthetic agent used plays a role in the disruption of sensory processing; Lamme et al. [76] reported that under a different anesthetic protocol (fentanyl/NO) figure-ground responses in V1 at least partially recovered. Bahmani et al. [78] recorded MUA and LFP in V1 in passive fixating and fentanyl anesthetized monkeys. In their flash suppression paradigm a monocular image is presented. After an adaptation period (1–2 s) a second image is added and presented monocularly to the other eye: The two eyes then receive dissimilar images. The second (flashed) image is the perceived image and the first image is undetected and thus suppressed despite sensory stimulation. Visual adaptation cannot explain the suppression by itself and an additional suppression mechanism is believed to act upon awareness. Under anesthesia with fentanyl MUA and LFP modulation reflecting the percept were maintained with reduced amplitude.

The main reason for our use of the opiate remifentanil (a μ-opioid receptor agonist) over more typical anesthetics such as isoflurane, desflurane, or propofol is that remifentanil has no significant effect on neurovascular coupling in visual cortex, outside the "pain matrix" [79]. Opiate receptor density varies across brain areas [80], but is highest in pain-matrix regions [81]: basal ganglia, thalamus, insula, cingulate cortex, somatosensory/motor cortices, as well as orbitofrontal, frontal and parietal cortices. These regions are typically active under noxious stimulation. The visual cortex, however, has low affinity for remifentanil [82]. The responsiveness of visual cortex to visual stimuli is opioid dose independent. However the activation of the pain matrix by pain stimuli does depend on the dose [83–86]. In contrast *isoflurane/xylazine* shows dose-dependent effects in visual pathways [77].

Goense and Logothetis [79] tested a similar anesthesia protocol and found that it did not disrupt the coupling between neural activity and the BOLD signal in V1. Ku et al. [87] studied extrastriate areas. When contrasting faces vs. objects they found BOLD activation in temporal, medial temporal and ventral temporal cortices. Interestingly, face selective activation was maintained during anesthesia in most areas. Activation of amygdala and hippocampus–areas typically involved in memory–was absent or ambiguous in anesthetized subjects.

Conscious perception may of course depend on processing outside V1, though in some situations the global percept of Glass patterns may occur even in the absence of awareness [88]. It is also notable that under our anesthetic conditions we are able to record BOLD in extrastriate areas during checkerboard stimulation in adults, showing that our regime does measure extrastriate BOLD activity.

### Signal variability

Although pinwheel activation in V1 was apparent at early ages (Fig 3), it is somewhat surprising that the youngest sessions did not yield consistent classification (Fig 7), even in stimulus versus blank for Glass patterns. Some of the youngest sessions did show *d’ > 2*, but our criteria for estimating the age at which the developmental function—gauged by a saturation fit—exceeded *d’ = 2* resulted in older classification ages. The session-to-session variation, across and within animals, highlights the fact that there are multiple sources of noise that potentially affect classification accuracies in this study. On the one hand, there is noise intrinsic to the process of imaging BOLD responses. On the other hand, noise is added as result of ongoing visual development and changing neural processes. Individuals develop at different rates, and “ages” used are post-natal rather than post-conceptional, so some session-to-session variance will be explained by these factors. Finally, since the brain and head are growing, it is conceivable that the cortical surface in infants is slightly different from that in adults. While the size of the brain does not substantially increase after 3 to 4 months, the white matter/gray matter ratio does continue to change over about 1.5 years [16]. An advantage of the MVPA is that accuracies were compared *within* a session; scan properties and cortical surfaces may have varied from session to session but the classifications were performed under equivalent conditions. By using identical computational techniques for all sessions and ages, we attempted to keep the intrinsic noise comparable across sessions and conditions. Therefore classification accuracies should reflect mainly the developmental processes and some animal-to-animal variation. Our saturation fitting-algorithm combines accuracy measures across multiple sessions and ages with the intent to control for such developmental differences.

### Comparison of fMRI with visual behavior

Previous psychophysical studies show that basic visual function is evident at birth in infant monkeys [14,89–92]. Global visual functions are also measurable in the early weeks and months after birth [26,68,93,94] although some abilities such as contour integration and Glass pattern perception show a delayed onset compared with others such as global motion perception. The visual functions most relevant to the current study are global motion and Glass pattern sensitivity. Global motion perception is evident before 10 weeks in macaques and Glass pattern sensitivity is demonstrated in most animals by 6 months [26,68]. Our fMRI BOLD results show that extrastriate areas V4 and MT-V5 reliably classify static versus dynamic Glass patterns only after two years of age. It seems that the appearance of reliable BOLD activity does not reflect the *onset or development* of visual ability. Rather, it correlates most closely with the *age at maturation* of visual ability since the development of both global motion and Glass pattern sensitivity becomes asymptotic around age 2 years in macaques.

The observed activation location in putative V1 of young subjects (Fig 4A) is suggestive of the far peripheral visual field. This could indicate visual edges driving BOLD more strongly than Glass patterns itself and voxels corresponding to a more central visual field remain subthreshold.

### Comparison of fMRI with neurophysiology

As discussed in the Introduction, single unit recordings show relatively adult-like receptive field properties early in development [9–12,19,20]. Nearly all measured properties are adult-like before 16 weeks after birth in macaques, although most behaviorally measured functions continue to develop at least up to 1 year of age [26,68,90,93]. The late development of fMRI BOLD, indexed by GLM or MVPA, clearly does not reflect the simple development of receptive field properties as measured by single cell recordings. The electrophysiological data are collected under comparable anesthetic conditions to our fMRI experiments, so the late functional development—reflected in reliable BOLD responses to Glass patterns—is unlikely due to weak or suppressed neural activity. However, BOLD response integrates over large populations of neurons [95]. Population responses are still developing in MT-V5 late after birth [10]. Therefore, it is possible that while individual cells encode visual stimuli in an adult-like fashion, the development of spatially localized networks of neurons with correlated activity is still taking place. Later developing feedback connections or extensive pruning mechanisms might control the formation of functional clustering. Indications of this kind of reorganization have been observed by Wattam-Bell and colleagues [96].

Because MVPA classification accuracy relies on local clustering, it will not be sensitive during the developmental phase if the organization is still immature. On the other hand, GLM analysis relies less on clustering, which could explain why MVPA classification emerges at later ages than positive GLM results.

An alternate consideration is that most analyses of neuronal response properties to date in infant visual cortex have focused on basic responsiveness and tuning properties. There are reports of somewhat delayed development of center/surround organization in V2 [19] and of sustained activity after the initial transient response in V1 and V2 in infants [12]. Some evidence suggests that the sustained activity arises from feedback from higher visual areas [97]. The reduction of the sustained activity could reflect an immaturity of feedback from higher visual areas while local feedforward organization is already adult-like. It is unclear exactly when feedback projections become adult-like in early visual areas. Although both feedforward and feedback projections are found to exist prenatally in nonhuman primates, it seems that forward and backward projections follow different developmental time courses and feedback connectivity continues to be remodeled postnatally [98–102].

A behavioral study of facilitation and suppression of stimulus detection by nearby stimuli [103] concluded that facilitatory lateral spatial interactions mature late, around age 1 year. Corroborating EEG data, from visual-evoked potentials [104], showed that lateral interactions are also not mature in young human infants. Perceiving the global form in Glass patterns depends on spatial integration of visual information. The ongoing development of lateral interactions might be reflected in a similarly late maturation of Glass pattern classification. Even if the intra- and inter-cortical connections are structurally similar to adults, unless physiological responses are also mature late development of reliable BOLD responses to Glass patterns could result.

### Dorsal and ventral stream development

We expected the “form” contrasts to reveal any distinction between the time courses for dorsal and ventral stream development. Unfortunately the contrast random vs. structured did not produce significant differential activity in any of the visual areas we studied. The GLM analysis yielded an earlier significant developmental trend for V4 than MT-V5 for the static vs. dynamic contrast, while the classification analysis converged on similar ages for V4 and MT-V5 with this contrast. Thus, although the BOLD response in both extrastriate areas emerged later than that in V1, there does not appear to be a reliable distinction between V4 and MT-V5. This conclusion is consistent with our behavioral study showing similar age at maturation for sensitivity to global motion and Glass pattern stimuli, which were designed to tap development of dorsal and ventral stream [26].

One might wonder whether we would have produced a different developmental profile for MT-V5 had we used real, or apparent, motion stimuli instead of dynamic Glass patterns. Human observers show lower thresholds for dynamic Glass patterns compared to static ones [105–107] despite the fact that dynamic Glass patterns contain no coherent “motion” signals [39]. Also, human and macaque observers show lower thresholds for global motion stimuli than for Glass patterns [26,107]. Single unit recordings [40] and fMRI in humans [41] reveal responses to dynamic Glass patterns in both MT-V5 and in V4, suggesting similar activation of dorsal and ventral stream.

In conclusion, this developmental study revealed relatively late appearance of robust fMRI BOLD activity to Glass pattern stimuli in infant macaque visual cortex. Our data support a hierarchical pattern of development from primary visual areas to higher order ones, but little distinction between areas at similar level, i.e., between V4 and MT-V5. This suggests parallel development of these dorsal and ventral stream areas as indexed by stimulation with Glass patterns. The development of BOLD activity correlated with the age at maturation of related psychophysical abilities rather than their physiological development per se.
